# Midazolam prescription as a trigger for potential deceased donor identification outside the ICU: a prospective feasibility study

**DOI:** 10.1186/s12904-026-02163-4

**Published:** 2026-05-29

**Authors:** J. A. Encarnación, B Domínguez-Gil, C. Pellicer, R. Fernández, J Moya, A. Ortin, C. Manso, M. Fernández, C. Lucas, R Jimena, N. López, J. L. Alonso-Romero, P. Ruiz, N. D. Ortega-López, M Royo-Villanova

**Affiliations:** 1 Hospital Clínico Universitario Virgen de la Arrixaca e Instituto Murciano De Investigación Biosanitaria, University of Murcia, Carretera Madrid-Cartagena s/n, El Palmar, Murcia, 30120 Spain; 2 Department of Radiation Oncology, Virgen de la Arrixaca University Clinical Hospital, Murcia, Spain; 3 Murcian Institute for Biomedical Research “Pascual Parrilla” (IMIB), Murcia, Spain; 4 Transplant Coordination Unit, Virgen de la Arrixaca University Clinical Hospital, Murcia, Spain; 5 National Transplant Organization, Madrid, Spain; 6 Department of Hospital Pharmacy, Virgen de la Arrixaca University Clinical Hospital, Murcia, Spain; 7 Department of Intensive Care Medicine, Virgen de la Arrixaca University Clinical Hospital, Murcia, Spain; 8 Department of Medical Oncology, Virgen de la Arrixaca University Clinical Hospital, Murcia, Spain; 9 Department of Internal Medicine, Virgen de la Arrixaca University Clinical Hospital, Murcia, Spain

**Keywords:** Midazolam, Donor Detection, Pharmaceutical Prescription, Electronic Health Records, Expanded Donor Pool, Palliative Care, Patient Autonomy.

## Abstract

**Background:**

Systematic identification of potential deceased organ donors outside the intensive care unit (ICU) remains challenging. Patients receiving palliative sedation are rarely evaluated for donation, particularly in non-critical care settings.

**Objective:**

To assess the operational feasibility of a semi-automated donor identification strategy triggered by midazolam prescription for end-of-life sedation outside the ICU.

**Methods:**

We conducted a 12-month prospective, single-center feasibility study in a tertiary university hospital in Spain. A daily pharmacy-based screening system identified new midazolam prescriptions outside critical care areas. The transplant coordination team reviewed alerts, assessed medical suitability, and explored donation preferences when appropriate. When consent was obtained, intensive care to facilitate organ donation was initiated within a controlled donation after circulatory determination of death (cDCD) pathway.

**Results:**

A total of 1,398 patients were screened (mean 3.8/day). Most alerts (94%) were excluded during rapid electronic review. Ninety-one patients underwent in-depth evaluation; 12 were medically suitable and 8 proceeded to cDCD, resulting in 19 transplanted organs. During the equivalent period in the preceding year, no referrals for organ donation originated from hospitalized palliative care services.

**Conclusions:**

A semi-automated system based on midazolam prescription is operationally feasible and can be integrated into routine hospital practice to support systematic donor identification outside the ICU.

## Introduction

The shortage of transplantable organs remains a major limitation for transplantation programs worldwide [1, 2]. To expand the donor pool, donor eligibility criteria have progressively broadened over recent decades, including older donors and selected comorbid conditions [3, 4].

Another strategy to expand the donor pool is intensive care to facilitate organ donation (ICOD). ICOD involves the initiation or continuation of intensive care measures in patients for whom further treatment is no longer considered therapeutically appropriate, with the aim of enabling posthumous organ donation within end-of-life care planning [5]. ICOD is increasingly used in controlled donation after circulatory determination of death (cDCD) pathways [6].

cDCD has become an important strategy for expanding deceased donor programs [2]. Potential cDCD donors include not only patients with devastating brain injury who do not meet neurological death criteria, but also patients receiving palliative and end-of-life care after withdrawal of active treatment [7]. However, many such patients, particularly outside intensive care units (ICUs), are never considered for organ donation because of operational and systemic barriers [8].

Spain, a global leader in organ donation [9], reported 2,562 deceased donors in 2024, corresponding to a rate of 54.6 donors per million population [10]. Nevertheless, given that nearly half a million deaths occurred in Spain that same year, fewer than 1% of deaths resulted in organ donation. One contributing factor is the failure to identify transitions from active treatment to palliative and end-of-life care as opportunities for considering organ donation. Timely identification of such situations would enable the application of ICOD, allowing for donation through cDCD pathways.

Donor identification typically relies on clinical teams to recognize and refer possible organ donors to transplant coordinators (TC) or the staff of organ procurement organizations [11]. Effective referral depends on structured protocols, continued training, feedback mechanisms, and adherence to routine referral policies. Protocols usually specify clinical triggers that should prompt donor referral by treating teams. Proactive strategies led by donor coordinators, such as regular clinical rounds and review of hospital admissions for signs of imminent death, are also essential [12]. However, these practices become increasingly complex when extended beyond the ICU setting and involving palliative care. To address these limitations, semi-automated systems that facilitate the regular identification of possible organ donors can reduce reliance on human vigilance and subjective judgment. One promising approach involves using pharmacologic end-of-life sedation, particularly with midazolam, as a trigger for early donor identification. Such systems could enhance consistency and uncover donation opportunities that might otherwise be missed.

The integration of organ donation into end-of-life care also raises important ethical considerations, particularly in patients receiving palliative sedation. While organ donation may represent an opportunity to respect patients’ values and altruistic wishes, the implementation of intensive care to facilitate donation and cDCD pathways may also modify the dying trajectory and introduce potential tensions between end-of-life care priorities and donation-related procedures. Ensuring that comfort, dignity, autonomy, and the primary goals of palliative care remain central throughout the process is therefore essential.

Despite increasing interest in semi-automated systems to support deceased donor identification, most existing approaches have focused on intensive care settings. Evidence regarding automated or semi-automated identification strategies applicable to patients receiving palliative and end-of-life care outside the ICU remains scarce. In this context, the present study aimed to assess the operational feasibility of using midazolam prescription for end-of-life sedation as a pharmacological trigger to systematically identify potential deceased organ donors outside the ICU.

## Methods

This was a prospective, interventional, single-center study conducted at a tertiary university public hospital in Spain.

Each weekday morning at 08:30 AM, the hospital pharmacy department performed an automated computerized search to identify all new midazolam prescriptions issued within the previous 24 h outside critical care, intensive care, and post-anesthesia care units. These areas were excluded because midazolam is routinely used there for procedural sedation, postoperative recovery, and analgesia unrelated to end-of-life care, which would substantially reduce the specificity of the screening strategy. In our institutional setting, midazolam prescription outside critical care and perioperative settings is predominantly associated with palliative or end-of-life sedation [13, 14].

The resulting list of prescriptions was securely transmitted, using double-encrypted institutional email, to the transplant coordination team.

Following receipt of the automated alert list, a preliminary electronic screening was independently performed by members of the transplant coordination team experienced in donor evaluation. Electronic medical records were rapidly reviewed to identify patients with predefined contraindications to organ donation.

Exclusion criteria at this stage included age > 75 years, active malignancies considered absolute contraindications to donation according to national recommendations, uncontrolled infections, or other major medical contraindications to deceased organ donation. The upper age threshold was pragmatically selected for this pilot feasibility phase and was not intended to represent a definitive donor eligibility criterion and may have contributed to selection bias by excluding potentially suitable older donors.

Clinical records were additionally reviewed to verify that midazolam prescriptions corresponded to palliative or end-of-life sedation rather than alternative clinical indications.

This preliminary screening phase was generally completed between 08:30 AM and 10:00 AM and required less than one minute per patient in most cases.

Patients meeting the preliminary screening criteria subsequently underwent an in-depth multidisciplinary evaluation involving the transplant coordination team and the treating clinical team responsible for the patient’s care.

This evaluation included detailed assessment of prognosis, comorbidities, potential contraindications to donation, anticipated end-of-life trajectory, and the clinical and ethical appropriateness of considering organ donation within the context of ongoing palliative care. Discussions were also held with the treating team to ensure clinical consensus and appropriate integration of donation evaluation within routine end-of-life care planning.

Some patients had died overnight before completion of the transplant coordination assessment. Evaluations were generally conducted between 10:00 AM and 1:00 PM on weekdays. Due to logistical limitations, systematic evaluation could not be performed during weekends, public holidays, or afternoon hours.

When patients were considered potentially suitable for donation after multidisciplinary evaluation, donation-related discussions were conducted either with the patient, when decision-making capacity was preserved, or with the legal representatives according to the Spanish legal framework. Decision-making capacity was clinically assessed by the treating medical team before any donation-related discussion took place. Donation discussions were conducted directly with patients only when they were considered capable of understanding the information provided and expressing autonomous decisions regarding end-of-life care and organ donation.

Discussions were conducted by trained members of the transplant coordination team after introduction by the healthcare professionals responsible for patient care. Discussions followed the institutional framework routinely used by the transplant coordination team for end-of-life donation pathways. Information provided included the possibility of organ donation, ICU admission for intensive care to facilitate organ donation (ICOD), elective ventilation, donor evaluation procedures, withdrawal of life-sustaining treatment, and controlled donation after circulatory determination of death (cDCD), including both antemortem and postmortem procedures relevant to organ preservation and donation. Written informed consent was obtained in all cases in which ICOD and donation were accepted. Following acceptance of ICOD and provision of informed consent, patients were admitted to the ICU within an integrated end-of-life care plan that incorporated the expressed wish to pursue organ donation.

Sedation and analgesia were maintained exclusively to ensure comfort and dignity according to institutional palliative care protocols, independently of the donation process. Donation-related procedures performed during the ICU phase included standard donor evaluation, physiological monitoring, and organ preservation measures routinely applied within established cDCD protocols. Clinical management and withdrawal of life-sustaining treatment were performed according to standard institutional end-of-life care protocols and in accordance with national ethical and legal standards governing cDCD.

Once withdrawal of life-sustaining treatment was initiated and death was confirmed according to circulatory criteria, the cDCD process was undertaken following established national and institutional protocols.

### Descriptive historical contextualization

To provide contextual background regarding donor referral practices prior to implementation of the automated alert system, a retrospective descriptive review of hospital records from the equivalent calendar period of the preceding year was performed.

This exploratory review was limited to identifying whether referrals for deceased organ donation had originated from hospitalized patients receiving palliative or end-of-life care outside the ICU. No retrospective application of the automated screening strategy was performed, and no formal comparison with the prospective feasibility cohort was intended.

The historical review was therefore descriptive only and aimed exclusively to provide institutional context regarding baseline referral practices before implementation of the pharmacological alert system.

### Variables and analysis

Information was compiled on cases identified by the prescription of midazolam, cases reviewed by the donor transplant coordination (DTC) team, and those deemed medically suitable and unsuitable. Information was also collected on patients approached to explore donation opportunities and their transition to actual donation (at least one organ recovered). Reasons why potential donors did not evolve to actual donation were also recorded. Demographic and clinical features of actual deceased organ donors were described, along with information on organs recovered and transplanted.

We performed a descriptive analysis of the cases.

### Ethical considerations

The study protocol was reviewed and approved by the local clinical research ethics committee (Protocol No. 2024-12-7-hcuva). Written informed consent was obtained from all patients or their legal representatives, including authorization for the use of anonymized clinical data for research and publication purposes.

Within the framework of the protocol to evaluate patients as possible organ donors, interviews were conducted in accordance with applicable ethical and legal standards. These interviews were designed to provide clear information, convey empathy, and offer emotional support during a highly sensitive period. All discussions were led by properly trained members of the donor transplant coordination team.

All pharmacologic measures applied during end-of-life care, including sedation, adhered to established palliative care standards and were not influenced by the donation process.

## Results

During the 12-month study period, 1,398 automated pharmacological alerts based on new midazolam prescriptions were generated and reviewed by the transplant coordination team (mean 3.8 alerts/day).

During preliminary electronic screening, 1,314 patients were excluded, including 947 patients with active oncological diseases considered contraindications to donation and 367 patients aged > 75 years.

A total of 91 patients subsequently underwent in-depth multidisciplinary evaluation. Of these, 79 were excluded because of additional contraindications to donation, including uncontrolled infections, poor organ function, or other clinical factors considered incompatible with deceased organ donation.

Twelve patients were considered medically suitable and underwent complete donation discussions with patients or legal representatives. In four cases, donation was declined. Ultimately, eight patients proceeded to ICU admission for intensive care to facilitate organ donation and underwent controlled donation after circulatory determination of death (cDCD), resulting in 19 transplanted organs.

The preliminary electronic review generally required less than one minute per patient, whereas in-depth multidisciplinary evaluation required approximately 10–20 min per potentially suitable case.

In total, 19 organs (11 kidneys, 6 livers, 1 lung and 1 heart) were transplanted from these 8 donors, whose clinical characteristics were as follows:

- *Donor 1*: 44-year-old male diagnosed with Huntington’s disease who developed a progressive respiratory failure during hospital admission; donated two kidneys and liver.
- *Donor 2*: 66-year-old female with a history of cerebral hemorrhage three years before progressive functional decline; admitted for palliative sedation; donated two kidneys and liver.
- *Donor 3*: 65-year-old female with lifelong neurological impairment, admitted with pneumonia; donated two kidneys (liver deemed unsuitable).
- *Donor 4*: 75-year-old male with a five-year history of advanced dementia, admitted to the hospital from a long-term care facility with respiratory infection and agonal state; donated the liver.
- *Donor 5*: 56-year-old female with a long-standing history of advanced dementia, admitted with progressive functional decline and intercurrent infection; donated one kidney and lungs (liver considered unsuitable).
- *Donor 6*: 45-year-old male admitted to the neurosurgery unit with severe neurological deterioration secondary to spontaneous intracranial hemorrhage. Although catastrophic neurological injury was present, progression toward death by neurological criteria was not considered clinically likely at the time of evaluation, and the patient was therefore managed within a controlled donation after circulatory determination of death (cDCD) pathway; donated two kidneys, liver and heart.
- *Donor 7*: A 68-year-old male with an eight-year history of advanced dementia, admitted to the hospital from a long-term care facility with a urinary tract infection and in an agonal state; donated the liver.
- *Donor 8*: A 59-year-old female with a long-standing history of advanced dementia, admitted with progressive functional decline and intercurrent infection; donated the liver and both kidneys.

### Contextual historical findings

During the equivalent calendar period preceding implementation of the automated alert system, no referrals for deceased organ donation originating from hospitalized patients receiving palliative or end-of-life care outside the ICU were identified in institutional records.

## Discussion

The initial findings of our study suggest that semi-automated donor identification based on the prescription of medication for palliative sedation represents a feasible strategy for the systematic evaluation of potential donation opportunities outside the ICU. Traditionally, efforts to identify potential deceased organ donors have focused on the ICUs. In recent years, strategies to increase deceased donation have expanded to other hospital areas. When persons are in a situation of imminent death and the clinical team makes the decision to shift from active treatment to palliative and end-of-life care, exploring donation opportunities becomes critical not only to address the needs of organs for transplantation, but also to account for the overall interests of the dying patient. This double value of organ donation has recently been acknowledged by the World Health Assembly through Resolution 77.4, adopted in May 2024 [15]. Though the identification of possible organ donors outside the ICU has mainly been focused on patients with a devastating brain injury, cDCD now allows to consider a variety of clinical conditions where palliative sedation is applied. Patients will require ICOD to incorporate organ donation into their end-of-life care plans.

In some cases, particularly among patients with severe neurological injury, distinction between potential progression toward death by neurological criteria and planned cDCD pathways may not be absolute at the time of evaluation. In the present study, patients were managed within cDCD pathways when progression toward brain death was not clinically expected or sufficiently established to justify a donation after brain death (DBD)-based approach.

Donation outside the ICU is however hampered by the lack of training of palliative healthcare professionals in deceased donation, as well as the advanced age and comorbidities of patients, including a high prevalence of cancer. Though training and education by DTCs, established protocols with specified clinical triggers, and continuous education on routine referral can help to overcome these barriers, variability in the level of knowledge of professionals in charge of palliative care remains a challenge, underscoring the need for a more standardized approach. It is important to emphasize that this semi-automated system does not replace the need for specific training and continuous education of palliative care professionals in deceased organ donation. Rather, it should be understood as a complementary tool that supports clinical teams, facilitates timely identification of potential donors, and enhances collaboration with transplant coordination services, without diminishing the central role of professional judgment and expertise.

Traditional donor identification and referral is primarily based on clinical judgment and established clinical triggers (e.g. mechanical ventilation, low Glasgow Coma Score, or clinical condition consistent with brain death). However, this approach has been described to miss 30–60% potential organ donors [16]. Automated identification systems have shown to facilitate a timely referral of deceased organ donors [17, 18]. We proposed this same approach integrated into the pharmaceutical prescription system. Since the use of midazolam in patients under palliative care is a standard for managing refractory symptoms, in fact, it is the most frequently used benzodiazepine in our setting for end-of-life care and is recommended by the Spanish Society for Palliative Care, establishing such prescription as an objective pharmacological trigger, could support a more systematic and potentially less subjective identification process, particularly outside the ICU. To the best of our knowledge, donor detection has never been triggered by the prescription of this drug.

Our preliminary results, showing eight actual donors identified through the system in over a one-year period, demonstrate the feasibility of this approach. The contextual historical review provides additional insight into the potential relevance of this identification strategy. During the same calendar period in the year preceding implementation, no referrals for deceased organ donation originated directly from hospitalized palliative care services at our institution. In contrast, following the introduction of the semi-automated alert system, eight patients admitted under palliative care pathways proceeded to actual controlled donation after circulatory determination of death. Although this observation does not allow causal inference or formal comparison, it provides contextual support for the potential relevance of systematic donor identification strategies in this clinical setting where referrals are otherwise uncommon or absent.

It should be acknowledged that the implementation of intensive care to facilitate organ donation (ICOD) and controlled donation after circulatory determination of death (cDCD) is uneven across countries. In Spain, this practice is well established within a clear ethical and legal framework, supported by the National Transplant Organization (ONT) and national legislation. However, in other jurisdictions, ICOD may not yet be formally integrated into end-of-life care pathways, which could limit the external applicability of our model. International harmonization of ethical and procedural standards will be necessary to ensure that similar strategies can be safely and effectively adopted in diverse healthcare settings.

The strategy must be analyzed from a number of perspectives, including its impact upon the quality of end-of-life care. Organ donation can uphold patient autonomy in the context of palliative care. For some patients and families, organ donation may represent an opportunity to align end-of-life care with personal values and altruistic preferences. Allowing palliative patients to be evaluated as potential organ donors may introduce an additional dimension of altruism and personal legacy within end-of-life care discussions. However, donation must be presented in a respectful and sensitive manner to patients and their families, by appropriately trained professionals. Nevertheless, integrating organ donation into end-of-life care may also generate ethical tensions, particularly when intensive care interventions are introduced in patients already receiving palliative care. Careful separation between decisions regarding treatment limitation and decisions regarding donation remains essential to ensure that end-of-life care priorities, patient comfort, dignity, and autonomy are never subordinated to donation objectives. Donation-related interventions were only implemented after independent end-of-life decisions had been established by the treating teams according to standard clinical practice.

Donation discussions were integrated within routine end-of-life care and were conducted only after agreement between the treating clinical team and the transplant coordination team regarding the appropriateness of considering organ donation in each individual case. The approach aimed to ensure that palliative care objectives, including patient comfort, dignity, and respect for autonomy, remained central throughout the process.

Sedation and analgesia were administered strictly according to institutional palliative care protocols, with the sole purpose of ensuring patient comfort and dignity, independently of the donation process or its timing. This clarification is crucial to ensure that integrated care for potential donors fully aligns with ethical standards governing end-of-life care.

The project demonstrated the operational feasibility of implementing a structured system to identify and evaluate potential donation opportunities among patients receiving palliative care, by establishing a structured system to identify, evaluate, and potentially offer patients in palliative care the possibility of organ donation. The number of cases reviewed was large during the study period, but the mean was below four cases per day. The initial electronic review of each case, primarily focused on age and oncological status, took less than one minute per patient, while the in-depth evaluation of those meeting basic criteria (approximately 1–2 per week) required between 10 and 20 min. Therefore, the daily workload for the donor coordination team was minimal, and the strategy proved operationally sustainable.

For healthcare professionals in charge of palliative care, the system reduced the burden of systematically considering and evaluating donation opportunities and likely referring unsuitable cases. Though the system did not preclude the need for continuous education and training of these professionals, was designed to facilitate interaction between palliative care teams and transplant coordination services. The operational workload associated with the screening strategy appeared manageable within routine clinical practice. However, the algorithm could be refined to improve its accuracy and efficiency, accounting for additional information incorporated into hospital electronic health records. Moreover, a notable advancement could rely on the application of machine learning. Recently, Sauthier et al. developed a convolutional neural network model using longitudinal laboratory and clinical data to screen for potential organ donors [19]. The model achieved an area under the ROC curve of 0.966 (CI 0.949–0.981), with 84% sensitivity and 93% specificity, outperforming logistic regression models, especially for rare or complex cases.

Pharmacy Services within the hospital’s organizational structure are considered central services responsible for the management, logistics, and coordination of the entire medication circuit. This includes everything from inclusion in the pharmacotherapeutic guide, acquisition, protocolization in indication and medical prescription, validation and pharmacotherapeutic follow-up of the patient, to treatment administration, ensuring safety and quality at each stage in all hospital medical services.

The active involvement of the Pharmacy Services in this study represented an additional organizational and operational commitment beyond their routine functions. Daily searches for midazolam prescriptions, performed systematically at a fixed time each morning, required dedicated staff time and coordination with the donor transplant team, ensuring the accuracy and timely delivery of patient information. This proactive role may facilitate earlier identification of potential donors and support interdisciplinary coordination, positioning the pharmacy team as a key player in optimizing the donation process and contributing to the overall success of the study.

Further research is needed to validate these findings in larger-scale studies with diverse patient populations and to further analyze the impact of our approach on the abovementioned areas. Collectively, this project not only contributes to expanding the donor pool and the life expectancy of potential organ recipients but also establishes new opportunities for patients in palliative care units, allowing their wishes and values to be fulfilled by becoming organ donors. In medical practice, it promotes a more holistic, equitable, and collaborative vision of end-of-life patient care.

Recent evidence further supports the expansion of donor eligibility criteria. Older patients and those with renal dysfunction, traditionally excluded from donation, have demonstrated acceptable outcomes as deceased organ donors. Recent publications [20, 21] highlight that even expanded-criteria donors, including elderly individuals or those with renal impairment, can contribute successfully to transplantation programs, with graft survival rates comparable to standard donors in selected contexts and in patients with brain tumor. These findings reinforce the need to reassess conventional contraindications and broaden the donor pool in a safe and evidence-based manner.

## Limitations

Several limitations of this study should be acknowledged. First, this was a single-center feasibility study conducted within a healthcare system where ICOD and cDCD are well established, which may limit generalizability to other jurisdictions with different legal, ethical, or organizational frameworks. Second, the screening strategy relied on local prescribing practices, where midazolam use outside critical care settings is predominantly associated with palliative or end-of-life sedation. Applicability may therefore differ in other healthcare settings. Third, although the study evaluated operational feasibility, it did not include formal empirical assessment of clinical acceptability, healthcare professionals’ perceptions, family experiences, or ethical outcomes. The study also did not evaluate the diagnostic performance of the screening strategy, including sensitivity, specificity, or missed potential donor cases. Fourth, detailed quantitative information regarding whether donation discussions were conducted directly with patients or legal representatives was not systematically recorded during the feasibility phase and therefore could not be fully analyzed. In addition, the retrospective historical assessment was descriptive only and not directly comparable to the prospective feasibility phase. Finally, systematic evaluations were limited to weekdays and daytime hours, which may have resulted in missed potential donor identifications.

## Conclusion

**Fig. 1:**
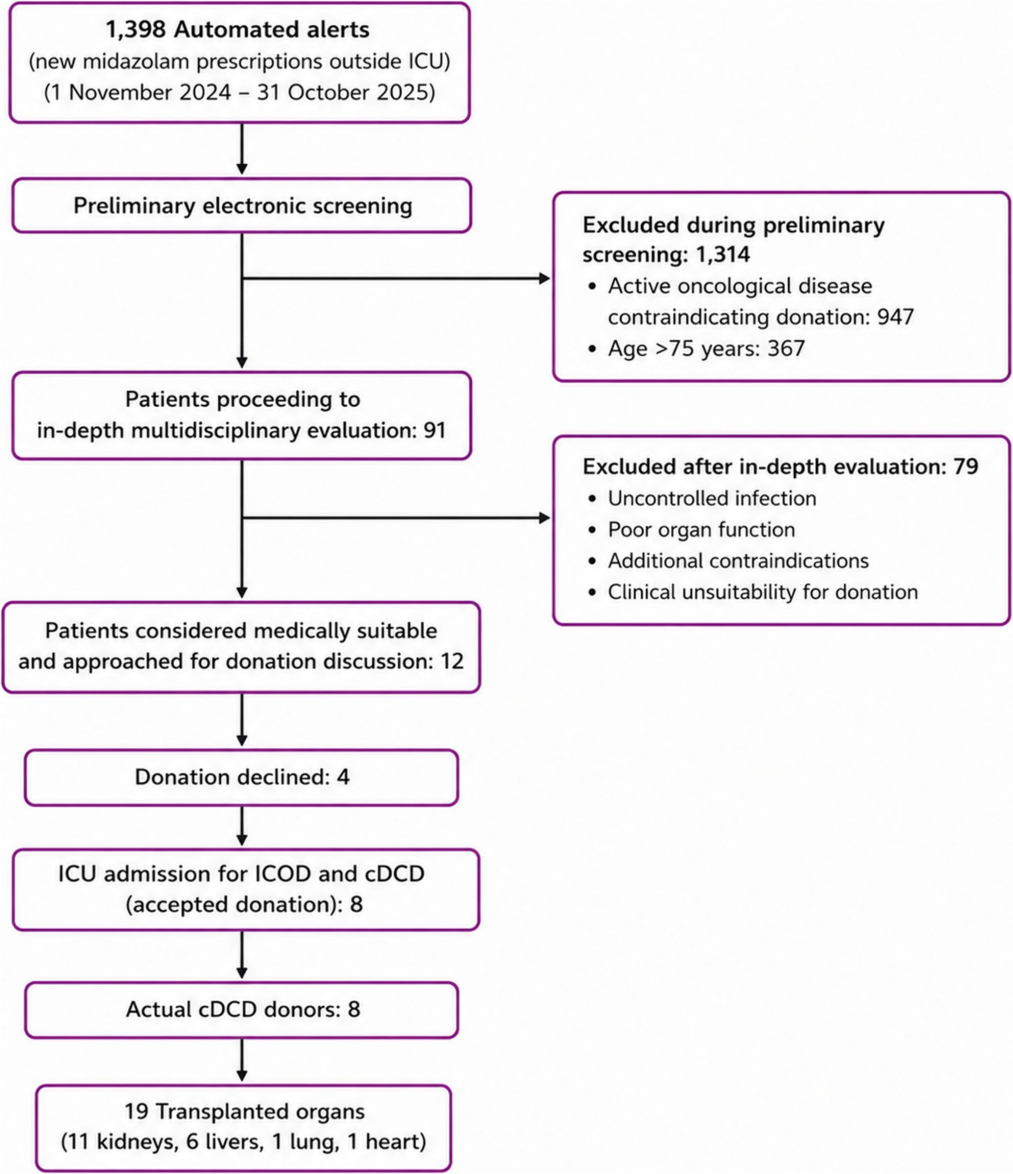
Flow chart of the evaluation process

Our findings suggest that a semi-automated donor identification system based on midazolam prescription for palliative sedation is operationally feasible in selected hospital settings outside the ICU. This approach may support more systematic evaluation of potential donation opportunities within end-of-life care pathways. Further multicenter studies are needed to assess its applicability, diagnostic performance, ethical acceptability, and integration across different healthcare systems (Fig. 1).

## Data Availability

The datasets generated and/or analyzed during the current study are not publicly available due to institutional data protection policies but are available from the corresponding author on reasonable request and subject to ethical approval.
